# The Impact of Advanced Glycation End-Products (AGEs) on Proliferation and Apoptosis of Primary Stem Cells: A Systematic Review

**DOI:** 10.1155/2020/8886612

**Published:** 2020-11-14

**Authors:** Lize Evens, Hanne Beliën, Dorien Deluyker, Annelies Bronckaers, Pascal Gervois, Marc Hendrikx, Virginie Bito

**Affiliations:** ^1^Biomedical (BIOMED) Research Institute, Hasselt University, Agoralaan Building C, 3590 Diepenbeek, Belgium; ^2^Faculty of Medicine and Life Sciences, Hasselt University, Martelarenlaan 42, 3500 Hasselt, Belgium

## Abstract

Stem cell-based regenerative therapies hold great promises to treat a wide spectrum of diseases. However, stem cell engraftment and survival are still challenging due to an unfavorable transplantation environment. Advanced glycation end-products (AGEs) can contribute to the generation of these harmful conditions. AGEs are a heterogeneous group of glycated products, nonenzymatically formed when proteins and/or lipids become glycated and oxidized. Our typical Western diet as well as cigarettes contain high AGEs content. AGEs are also endogenously formed in our body and accumulate with senescence and in pathological situations. Whether AGEs have an impact on stem cell viability in regenerative medicine remains unclear, and research on the effect of AGEs on stem cell proliferation and apoptosis is still ongoing. Therefore, this systematic review provides a clear overview of the effects of glycated proteins on cell viability in various types of primary isolated stem cells used in regenerative medicine.

## 1. Introduction

Regenerative therapies, including stem cell treatments, hold a high potential for treating patients with a spectrum of diseases. Stem cells can stimulate endogenous tissue repair mechanisms or replace damaged, necrotic tissue [1]. Stem cells are defined as undifferentiated cells with unlimited self-renewing capacity. They have the potential to form identical clones throughout the symmetrical division but can also differentiate into multiple cell types depending on the stem cell potency [1]. The source of stem cells is diverse as they can be found throughout the body in embryonic, fetal, and adult stages [2]. Because stem cells are the building blocks of organs and tissues, they are interesting candidates for regenerative medicine in order to repair multiple types of injuries [3]. For example, mesenchymal stem cells (MSCs) have the potential to differentiate into adipose, bone, or cartilage tissue, which makes them attractive candidates for the regeneration of these tissues in multiple diseases or injuries such as metabolic bone diseases or osteoarthritis [4–6]. Neural stem cells (NSCs) are adult precursor cells, therapeutically relevant in diseases of the brain and central nervous system, such as Alzheimer's disease or stroke [7]. Adipose tissue-derived stem cells (ADSCs), a specific type of MSCs isolated out of the adipose tissue [8], have been found to modulate inflammation, thereby promoting chronic wound healing [9]. Endothelial precursor cells (EPCs) are found in the bone marrow or blood and are capable of migrating towards lesions due to tissue ischemia or traumatic injury [10]. In addition, EPCs are involved in endothelial repair in patients with diabetes and atherosclerosis. Finally, blood-derived stem cells (BDSCs) are used in the clinic to restore the hematopoietic system in the blood and bone marrow malignancies or in autoimmune diseases [11]. However, despite their promising paracrine effects, differentiation, and migration capacities for repairing injured tissue, transplantation of stem cells remains challenging due to low cell engraftment, low cell survival, and suboptimal transplantation conditions [12]. Oxidative stress, presence of inflammatory cytokines, and/or advanced glycation end-products (AGEs) contribute to the generation of the harmful environment in which stem cells need to be transplanted and survive.

AGEs are a heterogeneous group of glycated proteins. They are formed when reducing sugars or aldehydes nonenzymatically react with proteins and lipids during posttranslational modifications [13]. Highly reactive dicarbonyl compounds, such as glyoxal or methylglyoxal, which can be oxidized by aldoses and ketoses, are also AGEs precursors [14]. AGEs can be endogenously formed in our body during hyperglycemia via the Maillard reaction and in situations of increased oxidative stress, such as increased levels of hydroxy radicals or decreased antioxidants, typically seen in an injured tissue. In addition, AGEs can also be absorbed via dietary compounds, especially when food is rich in both fat and proteins or cooked at high and dry heat [15].

Based on their molecular weight, AGEs are categorized in 2 classes: low-molecular weight AGEs (LMW-AGEs) and high-molecular weight AGEs (HMW-AGEs). There is no clear boundary between LMW-AGEs and HMW-AGEs. Gerdemann et al. defined LMW-AGEs as proteins with a molecular mass lower than 12 kDa [16], while HMW-AGEs are molecules with a molecular mass higher than 12 kDa. HMW-AGEs are considered to be protein-bound molecules which can form crosslinks, while LMW-AGEs tend to be free proteins or noncrosslinking. As for HMW-AGEs, high levels of LMW-AGEs like N(6)-carboxymethyllysine (CML), pentosidine, and pyraline are associated with different disease settings like diabetes, neurodegenerative diseases, and cardiovascular diseases [13, 17, 18]. The deleterious effects of AGEs throughout the body are classified according to their different mechanisms of action.

Firstly, AGEs can bind to specific cell surface receptors, e.g., receptor for AGEs (RAGE), and cause production of reactive oxygen species (ROS) and inflammatory cytokines or activation of intracellular pathways [19]. RAGE is a transmembrane receptor, localized on various cell types [20]. This receptor contains a binding site for various ligands, such as AGEs, high-mobility group protein box-1 (HMGB1), and members of the S100 protein family [21]. The full length of RAGE is anchored in the cell membrane with a transmembrane domain and contains an intracellular domain for signal transduction. Due to alternative splicing, several splice variants of RAGE are known in humans [22]. Soluble RAGEs (sRAGE) and endogenous secretory RAGE (esRAGE) are isoforms which are not anchored to the cell membrane. These variants lack the transmembrane domain and are therefore circulating forms, unable to be involved in signal transduction. They contribute in regulating and scavenging circulating ligands like AGEs [23]. Other splice events can lead to changes in the ligand binding domain of RAGE or the lack of the intracellular signal domain. Therefore, different splice variants can have different functionalities.

Next to binding to RAGE, AGEs can form crosslinks with proteins within the cell such as intracellular domains of different receptors or with proteins from the extracellular matrix such as collagen [24], leading to altered structural and functional properties of these proteins and thus organ function.

In the process of aging, AGEs contribute to decreased vessel elasticity, loss of skin plasticity, and degeneration of cartilage, ligaments, or the eye lens [25]. Furthermore, it has been shown that AGEs have an important role in the pathophysiology of different complications of diabetes mellitus, e.g., cardiomyopathy, retinopathy, neuropathy, and nephropathy [26]. Additionally, in cardiovascular diseases [27], Alzheimer's disease, and cancer, AGEs have been proven to display a causative role [28]. They can accumulate throughout the body in various tissues such as in the heart [29], blood vessels [30], lungs [31], and adipose tissue [32], exerting long-term effects.

Whether AGEs have an impact on stem cell viability and in situ proliferation in regenerative medicine remains unclear. Therefore, this systematic review provides an overview of the effects of glycated proteins on cell viability, proliferation, and apoptosis in various types of primary isolated stem cells. Unraveling the deleterious effects of AGEs on stem cells can help to tackle this issue in the future and may contribute to improved efficient stem cell therapy regenerative medicine.

## 2. Methods

### 2.1. Literature Search Identification

The primary objective of this systematic review was to assess the impact of AGEs on viability and proliferation of different primary stem cell types, by identifying the PICO elements (P = population: primary stem cells, I = intervention: AGEs, C = comparison: to control, and O = outcome: cell viability, proliferation, or apoptosis) [33]. In this systematic review, databases were searched for articles published from inception until the 27^th^ of October 2020. The electronic databases PubMed and Web of Science were used with the following mesh terms ‘Glycation End Products, Advanced' OR ‘Stem Cells' and the following keywords: (Advanced Glycation End Product OR Advanced Glycation End Products OR Advanced Glycation End-Product OR Advanced Glycation End-Products OR Advanced Glycated End Product OR Advanced Glycated End Products OR Advanced Glycated End-Product OR Advanced Glycated End-Products OR Glycated Protein OR Glycated Proteins) AND (Stem Cell OR Stem Cells OR Progenitor Cell OR Progenitor Cells).

### 2.2. Inclusion and Exclusion Criteria

After database searching in Pubmed and Web of Science, 339 abstracts of articles were included in the screening procedure. Articles were excluded based on different criteria: (1) articles with AGEs, RAGE, or stem cells as outcome measurements; (2) effects on other cell types than stem cells or progenitor cells; (3) articles about RAGE or diabetes and not AGEs; (4) articles with AGEs used as a diabetic model; (5) reviews; (6) book chapters; (7) announcements; (8) retracted papers; and (9) articles written in other languages than English. 75 full-text articles were assessed for eligibility. When experiments were not performed on primary stem cells, but on stem cell lines or when stem cells are provided and material and methods lack isolation procedure, articles were excluded. When outcome measurements were different from viability, proliferation, or apoptosis, articles were also excluded. Finally, 37 studies were included in this review.

### 2.3. Data Extraction, Analysis, and Quality Assessment

**Figure 1:**
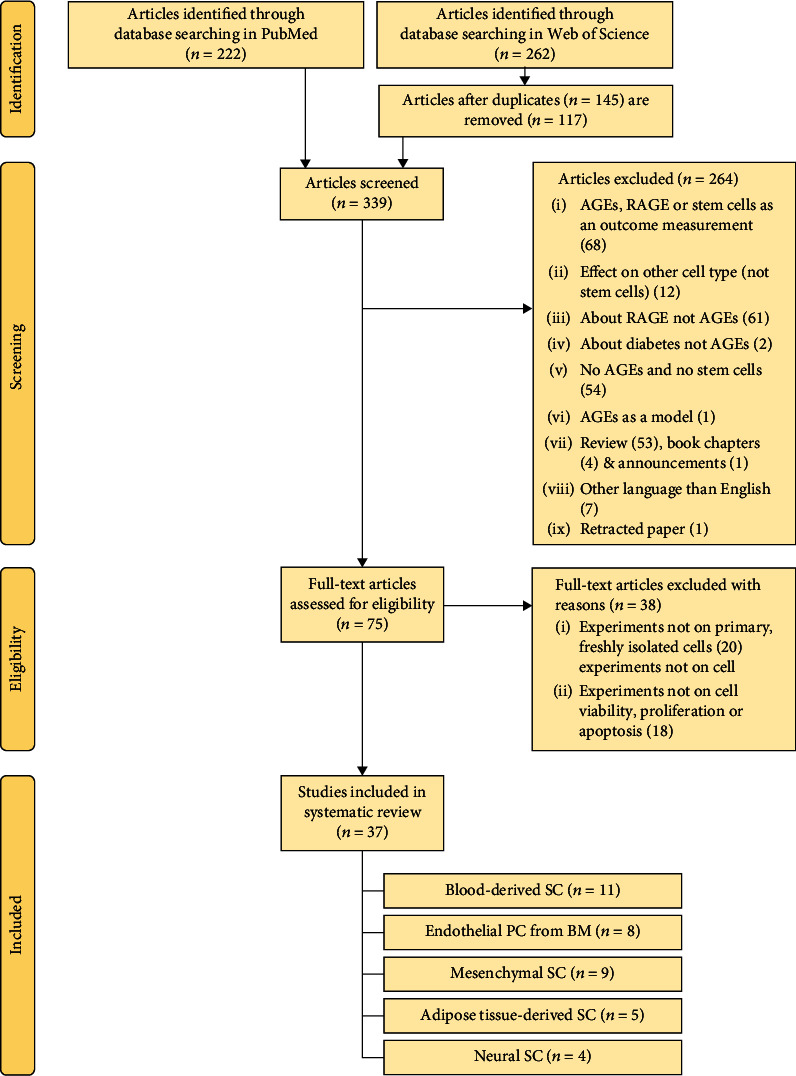
Flowchart summary of the search and selection of the included articles. AGEs: advanced glycation end-products; RAGE: receptor for AGEs; PC: progenitor cells; SC: stem cells; BM: bone marrow.

Results of the search were manually screened and are shown in Figure 1. Literature searches were independently performed by two reviewers (LE and HB). In case of disagreement, a consensus-based decision was made by the two reviewers to include/exclude an article. Data about the effect of AGEs on proliferation and apoptosis of different types of primary stem cells were analysed. Data were grouped based on stem cell type/isolation source: blood-derived stem cells (Table 1), endothelial progenitor cells from the bone marrow (Table 2), mesenchymal stem cells (Table 3), adipose tissue-derived stem cells (Table 4), and neural stem cells (Table 5). Because there is no standardized protocol for quality assessment for *in vitro* studies, the study quality of the selected full-text articles was assessed by two reviewers (LE and HB). When both reviewers judged the quality of study design to be inappropriate, articles were removed. Due to the high heterogeneity of the data (i.e., outcome measures, AGEs exposure duration, AGEs concentration, and experimental protocols), a meta-analysis could not be generated.

## 3. Results

### 3.1. Study Selection and Flow Diagram

The electronic databases PubMed and Web of Science were used to identify all articles regarding the impact of AGEs on primary stem cell proliferation and apoptosis. Study selection and flow chart diagram are shown in Figure 1. 222 and 262 articles were identified through database searching in PubMed and Web of Science, respectively. 145 duplicates were removed ending up to 339 articles being screened. 264 articles were excluded after screening. Then, 75 full-text articles were assessed for eligibility, resulting in the exclusion of 38 more articles. 20 articles about stem cells derived from cell lines or provided stem cells (with methods of isolation lacking) and 18 articles in which experiments about stem cell viability, proliferation, or apoptosis were not assessed, were excluded. 37 studies were therefore included in this review.

### 3.2. Study Results

#### 3.2.1. Blood-Derived Stem Cells

Blackburn et al. [34] investigated the effect of AGEs on peripheral blood mononuclear cells (PBMCs, Table 1). PBMCs were isolated from peripheral blood samples of healthy humans and cultured on 1 mM methylglyoxal- (MGO-) modified collagen gels for 4 days. Culturing PBMCs on MGO-modified collagen gels led to a decrease in cell number. In addition, endothelial progenitor cells (EPCs), a specific type of PBMCs, were investigated [1]. Isolation of EPCs from blood samples was performed by gradient density centrifugation [35–43] (Table 1). Regarding the origin of blood, one study isolated EPCs from umbilical cord blood [37] while in all other studies peripheral blood was used [35, 36, 38–44]. The concentration of AGEs application ranged from 2 *μ*g/ml to 400 *μ*g/ml. In addition, stimulation time varied from 1 up to 7 days. The effect of AGEs on EPCs from the blood are inconsistent. Bhatwadekar et al. [35], Chang et al. [36], Chen et al. [44], Li et al. [38], Liang et al. [39], Shen et al. [41], and Sun et al. [42] reported a decrease in cell proliferation and/or an increase in apoptosis of EPCs after exposure to AGEs. As opposed to these findings, Scheubel et al. [40] observed an increased EPCs proliferation after stimulation with a low dose of AGEs (20 *μ*g/ml). However, at higher concentrations (200 *μ*g/ml), AGEs caused a decrease in proliferation together with an increase in apoptosis. Zhu et al. [43] reported AGEs to have no effect on EPCs apoptosis but caused a decrease in proliferation, while Chen et al. [37] observed no effect of AGEs on EPCs proliferation but an increase in EPCs apoptosis. Concisely, compared to control conditions, all studies show that AGEs alter proliferation or stimulate apoptosis in EPCs derived from the blood.

#### 3.2.2. Endothelial Progenitor Cells Isolated from Bone Marrow

Isolating EPCs from the bone marrow is a standardized procedure [45–52] (Table 2), in which the tibia or femur of rodents (rats or mice) were flushed with media or PBS. AGEs were applied to EPCs in a fixed [46, 49] or dose-dependent [45, 47, 48, 50] manner, with concentrations ranging from 50 to 500 *μ*g/ml. The EPCs exposure time of 24 hours was the same in all studies, except for Zeng et al. [51], in which EPCs were stimulated for 48 hours. Wang et al. [52] stimulated in a time-dependent manner up to 48 hours. An increased apoptosis [16–18, 20–22] associated or not with a decrease in cell proliferation [16–19, 22, 23] as a result of AGEs exposure was reported in these studies. In short, AGEs negatively impact cell proliferation and increase apoptosis of EPCs isolated from the bone marrow.

#### 3.2.3. Mesenchymal Stem Cells

Nine articles have studied the effect of AGEs on mesenchymal stem cells (MSCs, Table 3) derived from BM, tendons, periodontal ligament, or the pancreas. Despite differences in the concentration (25 up to 800 *μ*g/ml) and duration (6 hours up to 19 days) of AGEs exposure, a decrease in proliferation associated or not with an increase in apoptosis, was observed in 7 out of 9 studies [53–60]. In contrast, Sakamoto et al. [56] observed a trend of decreased proliferation of MSCs by AGEs, but results did not reach significance. Duruksu and Aciksari [61] investigated MSCs isolated from pancreatic islet explants and cultured the cells on plates coated with modified collagen. In contrast with other studies, pancreatic MSCs showed an increase in proliferation when cultured on AGEs-modified collagen.

#### 3.2.4. Adipose Tissue-Derived Stem Cells

Five publications reported the effect of AGEs on adipose tissue-derived stem cells (ADSCs, Table 4). ADSCs are a type of MSCs, isolated from adipose tissue samples of humans [62–64], mice [65], and rats [66] by enzymatic dissociation with collagenase. Irrespective of the differences in concentration (20 up to 1600 *μ*g/ml) and duration (8 hours up to 7 days) of AGEs application, Li et al. [62] and Wang et al. [63, 64] reported an increase in apoptosis. Li et al. [65] and Zhang et al. [66] reported a decrease in proliferation. Taken together, AGEs have deleterious effects on ADSCs viability.

#### 3.2.5. Neural Stem Cells

Neural stem cells (NSCs) were investigated in four articles (Table 5). Brain tissues were isolated from rats to obtain cultures of proliferative neurospheres. Fleitas et al. [67] cultured NSCs for 6 days with 50 *μ*M MGO or glyoxal- (GO-) modified proteins. Meneghini et al. [68] and Wang et al. [69, 70] applied AGEs in concentrations ranging from 25 to 400 *μ*g/ml for 3 up to 7 days to NSCs. Fleitas et al. [67] observed apoptosis in NSCs due to modified proteins. The articles from Wang et al. [69, 70] reported a decrease in proliferation of NSCs after AGEs application, while Meneghini et al. [68] observed an increased proliferation.

## 4. Discussion

### 4.1. Effect of AGEs on Primary Stem Cell Proliferation and Apoptosis

According to this systematic literature review, AGEs cause a significant decrease in proliferation or an increase in apoptosis of BDSCs, ADSCs, and EPCs. In MSCs, a reduced stem cell viability was observed in 8 out of 9 studies. In NSCs, we can conclude that glycated proteins induce a decrease in proliferation or increase in apoptosis in 3 out of 4 articles. In short, our study reveals that AGEs are deleterious and alter the proliferative capacity of primary isolated stem cells in 35 out of 37 articles.

Compared to the results of other studies examining the effect of AGEs on MSCs, the controversial results of Duruksu and Aciksari [61] are likely due to the low concentrations of AGEs products used, i.e., 10 *μ*g/cm^2^-modified collagen, while the concentration of AGEs in other *in vitro* studies is generally ranging up to 500 *μ*g/ml. This was confirmed by Scheubel et al. [40] and Wang et al. [52]. Low concentrations of AGEs induce cell proliferation in EPCs, whereas at higher concentrations, they decrease the proliferative capacity of these cells. The concentrations of AGEs used in several *in vitro* studies are varying but generally reflect the physiological concentration of AGEs found in samples of patients. Indeed, AGEs-albumin concentration in diabetic patients has been shown to range from 50 to 400 *μ*g/ml [71, 72]. In patients suffering from cardiovascular diseases, AGEs levels can rise to concentrations up to 200 *μ*g/ml [73]. Other studies report lower AGEs concentrations *in vivo* in the range of several ng/ml, for example, in patients with early-stage Alzheimer's disease [74]. However, estimation of reliable AGEs concentrations *in vivo* is challenging, because of the heterogeneity of different types of AGEs and the different analytical methods used for measuring these AGEs [75]. Therefore, investigating a broad range of AGEs concentrations *in vitro* is necessary. Furthermore, in *in vitro* experiments, stem cells are exposed to AGEs in short term, while in several diseases, stem cells are exposed to AGEs for months or years. Therefore, subjecting these cells to higher concentrations of AGEs *in vitro* compared to *the in vivo* situation remains relevant [48].

In the same line of controversial results, Meneghini et al. [68] reported an increase in NSC proliferation after AGEs application, with concentrations ranging from 25 to 100 *μ*g/ml. A possible explanation for these controversial results could be, as stated in their article, that AGEs and other ligands of the RAGE receptor like HMGB1 and S100 calcium-binding protein B, are enhancing stem cell proliferation. Due to traumatic or ischemic brain injury, the binding of these specific ligands to the RAGE receptor can activate the NF-*κ*B signaling pathway, thereby inducing endogenous repair [68]. By increasing stem cell proliferation via the NF-*κ*B axis, damaged neurons and glia cells are replaced to repair the injured regions after brain injury. In line with these findings and hypothesis, Romanko et al. [76] and Jin et al. [77] also reported that neural progenitor cells in the subventricular zone proliferate and replace damaged neural cells after moderate brain insults.

### 4.2. Mechanisms Involved in the Decreased Proliferation or Increased Apoptosis

#### 4.2.1. AGEs Activate the Intrinsic and Extrinsic Apoptosis Pathways

**Figure 2:**
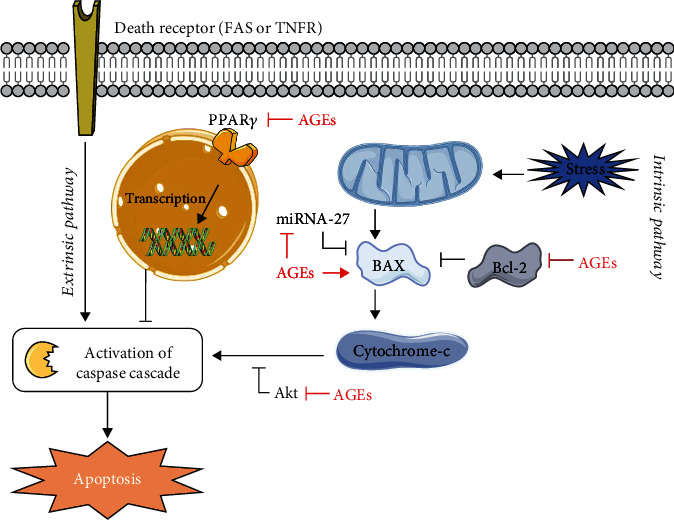
Interference of AGEs in the extrinsic and intrinsic apoptosis pathways. Via the extrinsic as well as intrinsic pathways, AGEs lead to an increase in apoptosis. AGEs release the blockage of PPAR*γ* on the caspase cascade. In addition, AGEs reduce the expression of miRNA-27 and Akt, increase the expression of BAX protein whereas the antiapoptotic Bcl-2 is inhibited, all resulting in an increase in apoptosis.

How AGEs interfere with the various apoptosis pathways is depicted in Figure 2. Wang et al. [69, 70] reported a decrease in proliferation of NSCs via PPAR*γ* inhibition. PPAR*γ* is responsible for blocking the caspase cascade in both the extrinsic and intrinsic apoptosis pathways (Figure 2) [78]. AGEs downregulate PPAR*γ* protein expression, which causes a release of caspase blockage, resulting in apoptosis stimulation. AGEs can interact with RAGE in order to activate multiple cellular signaling cascades, including MAP kinase (MAPK) pathways [79]. PPAR*γ* phosphorylation is therefore increased, resulting in a decrease of PPAR*γ* transcriptional activity. Indeed, it has been shown that PPAR*γ* agonists like rosiglitazone [39] or pioglitazone [59], added to *in vitro* cultures of EPCs and MSCs, respectively, reverse the deleterious effects of AGEs via PPAR*γ* activation. This is also confirmed in other cell types like chondrocytes [79], macrophages, or endothelial cells [80].

Apoptosis can also be induced via the intrinsic mitochondrial pathway (Figure 2) [78]. Bax, a proapoptotic, and Bcl-2, an anti-apoptotic regulatory protein, are involved in this pathway. Li et al. [49] identified that this intrinsic pathway was activated in MGO-stimulated EPCs via the reduction of miRNA-27. miRNA-27 is antagonizing this intrinsic apoptosis pathway. If AGEs downregulate miRNA-27, the apoptotic pathway is stimulated in an indirect manner. These data were confirmed by Jin et al. [47]. Another indirect way of inducing apoptosis is via Akt signaling. Chen et al. [37] showed that AGEs downregulate Akt, which is normally responsible for the inhibition of caspase activation [81]. AGEs exposure can also lead to increased Bax expression and to a reduction of Bcl-2 expression, stimulating apoptosis in EPCs [44].

#### 4.2.2. RAGE Activation Leads to MAP Kinase Activation and Generation of ROS

**Figure 3:**
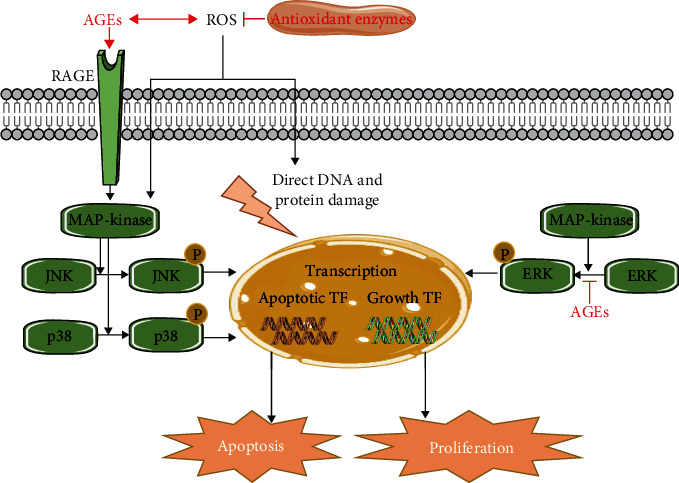
Interference of AGEs in the MAPK pathways. AGEs lead to an increase in apoptosis or a decrease in proliferation via the MAPK pathways. RAGE activation by AGEs causes activation of MAPK, which leads to phosphorylation of JNK and p38. These phosphorylated proteins increase the transcription of different proapoptotic transcription factors (TF), leading to an increase in apoptosis. Next to that, AGEs inhibit the phosphorylation of ERK, which normally promotes the transcription of growth factors leading to proliferation. Finally, AGEs also induce ROS formation by reducing the availability of antioxidant enzymes, which directly leads to DNA and protein damage. Indirectly, ROS interferes in the JNK/p38 MAPK pathway.

Figure 3 shows how activation of RAGE can induce apoptosis and reduce proliferation through the activation of several MAPK pathways. Zhang et al. [66] and Wang et al. [63, 64] reported activation of the AGEs/RAGE signaling pathway in ADSCs after exposure to AGEs. Binding of AGEs to their receptor RAGE activates the JNK and p38/MAPK pathways (Figure 3). Phosphorylation of JNKs and p38 causes upregulation of proapoptotic transcription factors in the nucleus, leading to an increase in apoptosis [82, 83]. In EPCs [41, 42, 46] and MSCs [53, 54, 58, 60], JNK/MAPK pathways are also activated by AGEs, leading to an increase in apoptosis. In addition, AGEs activate the MAPK pathways via excessive ROS generation. AGEs can decrease the availability of antioxidant enzymes, leading to increased oxidative stress [36, 38, 45]. Furthermore, RAGE activation by AGEs can directly induce activation of NADPH oxidase, leading to formation of ROS [84]. Next to the damaging effects of ROS on DNA and proteins, oxidative stress can also be a trigger for activating apoptosis via the JNK and p38/MAPK pathways. In MSCs and EPCs, excessive ROS production is thought to be responsible for the inhibitory effect of AGEs on stem cell proliferation [51, 55, 57].

Zhu et al. [43] investigated the effect of AGEs on the ERK/MAPK pathway. The ERK/MAPK pathway, in contrast to the JNK and p38/MAPK pathways, is responsible for cell growth (Figure 3) [85]. Activation of ERK via phosphorylation causes translocation to the nucleus where it induces transcription of factors related to cell growth and proliferation [86]. Zhu et al. [43] reported that AGEs caused less activation and phosphorylation of ERK, leading to decreased activation of growth transcription factors, resulting in a reduced proliferation.

#### 4.2.3. AGEs Induce Changes in Extracellular Matrix Composition and Stem Cell Attachment

Blackburn et al. [34] suggest that changes in extracellular matrix (ECM) proteins play a key role to induce stem cell dysfunction. AGEs-modified ECM has been shown to support less adhesion and retention of the stem cells, thereby causing detachment of stem cells which results in cell death [34]. In addition, the inhibitory effect of AGEs on EPCs derived from blood is also due to the modification of cell attachment and decreased capacity to adhere [40]. A possible underlying mechanism is that, at the site of injury, the recruited progenitors need to adhere to the preexisting vascular cells. AGEs block the RGD domain, a peptide sequence which is recognized by cell surface integrins. Therefore, EPCs cannot attach, spread, or migrate, leading to a decrease in their proliferative capacities [35].

#### 4.2.4. Alternative Signaling Pathways Affected by AGEs

According to Fleitas et al., [67] AGEs inhibit the processing from probrain-derived neurotrophic factor (pro-BDNF) to mature BDNF. BDNF is involved in neurotropic signaling including differentiation, survival, and synaptic plasticity of various populations of nerve cells, involved in tissue repair. Therefore, an increase in AGEs possibly cause an increased pathogenicity.

In addition, activation of RAGE can lead to the synthesis of miRNAs in stem cells known to regulate apoptotic signaling via intracellular ROS production. Li et al. [62] have found that miR-5591-5p is upregulated in ADSCs, when stimulated with AGEs. In the future, more insights on miRNA-mediated effects on stem cells is necessary, as it has been shown that microRNAs are key regulators in self-renewal processes in different types of stem cells [87].

### 4.3. Different Strategies to Improving Viability of Stem Cells by Tackling AGEs

**Figure 4:**
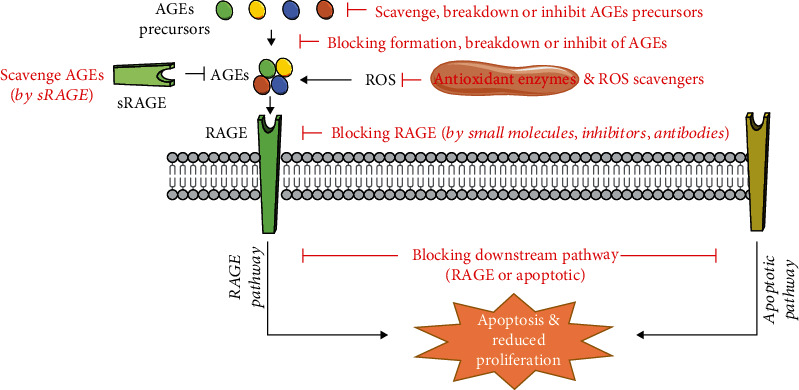
Strategies to tackle the effect of AGEs (88). AGEs lead to an increase in apoptosis or a decrease in proliferation via different pathways. This process can be tackled by different cellular approaches including scavenging, breaking down, or inhibiting AGEs and their precursors. RAGE can be directly blocked, or the downstream effectors can be inhibited. Oxidative stress can be reduced by antioxidants and ROS scavengers.

AGEs have detrimental effects on the viability of various primary stem cell types. However, tackling the deleterious effects of AGEs on stem cells is until now neglected but could potentially improve stem cell retention and viability. This could be achieved by several strategies, suggested by several studies [88], which are summarized in Figure 4. A first option is blocking RAGE with different synthetic small molecules [89], RAGE inhibitors such as FPS-ZM1 [90] or anti-RAGE antibodies [91]. Consequently, downstream pathways in the RAGE axis are not activated. Thereafter, directly blocking proteins involved in the apoptotic or RAGE pathway could be a way to interfere in the molecular pathways activated by AGEs. For example, MAPK can be targeted in order to block cellular signaling [92]. However, clinical trials reveal issues which relate to limited drug efficacy and toxicity of these compounds. AGEs and their precursors can also be directly inhibited or scavenged [93]. By increasing sRAGE, AGEs are trapped, RAGE is not activated, and the mediated signaling is attenuated. In addition, AGEs can also be broken down or AGEs formation can be suppressed [94]. Finally, ROS scavengers or antioxidants like N-acetylcysteine (NAC) can reduce oxidative stress levels and might interfere in the AGEs pathway. By these interventions, stem cell properties and viability could be improved. Such approaches require scientific proof but could open new therapeutic insights into stem cell transplantation as an effective regenerative therapy.

## 5. Conclusion

AGEs are increased in a lot of pathological situations and have detrimental effects on various tissues and cell types. In this systematic review, we show that AGEs impair the proliferation and apoptosis on different types of primary stem cells *in vitro*. These effects can be executed throughout several underlying mechanisms such as activation of RAGE or apoptotic pathways and excessive ROS generation. In the future, tackling this negative impact of AGEs on stem cells could improve stem cell properties, retention, and viability. Such approaches require solid scientific proof but could open new therapeutic insights into stem cell transplantation as an effective regenerative therapy.

## Figures and Tables

**Table 1:** Summary of included studies using BDSCs. Summary of isolation procedure and sampling, AGEs concentration, application duration, and effect on outcome measurement proliferation and apoptosis.

Study name	Year	Isolation SC/PC	AGEs application		Effect on outcome	
			Concentration	Duration	Proliferation	Apoptosis
Bhatwadekar et al. [35]	2008	Human EPCs–peripheral blood, DGC	Fibronectin coated with 10, 50, & 100 *μ*M MGO	24 hours	↘	
Blackburn et al. [34]	2017	Human PBMC–peripheral blood, DGC	Collagen type I gel +1 mM MGO	4 days	↘	
Chang et al. [36]	2017	Human EPCs–peripheral blood, DGC	500 *μ*g/ml	24 hours	↘	
Chen et al. [37]	2009	Human EPCs–umbilical cord blood, DGC	50, 100, 200, & 400 *μ*g/ml	24 hours	-	↗
Chen et al. [44]	2019	Human EPCs–peripheral blood, DGC	200 *μ*g/ml	48 hours	↘	↗
Li et al. [38]	2016	Human EPCs–peripheral blood, DGC	50, 100, & 200 *μ*g/ml	24, 48, and 72 hours	↘	
Liang et al. [39]	2009	Human EPCs–peripheral blood, DGC	50, 100, & 200 *μ*g/ml	7 days	↘	↗
Scheubel et al. [40]	2006	Human EPCs–peripheral blood, DGC	2, 20, & 200 *μ*g/ml	7 days	Low conc. ↗, high conc. ↘	↗
Shen et al. [41]	2010	Human EPCs–peripheral blood, DGC	2, 20, & 200 *μ*g/l	24, 48, and 72 hours	↘	↗
Sun et al. [42]	2009	Human EPCs–peripheral blood, DGC	200 *μ*g/ml	24 hours		↗
Zhu et al. [43]	2012	Human EPCs–peripheral blood, DGC	15 to 3704 *μ*g/l or 250 to 1000 *μ*g/l	24, 48, and 72 hours	↘	-

SC: stem cell; PC: progenitor cells; ↘: decrease; ↗: increase; -: no effect; EPCs: endothelial PC; PMBC: peripheral blood mononuclear cell; DGC: density-gradient centrifugation; MGO: methylglyoxal.

**Table 2:** Summary of included studies using EPCs isolated from the bone marrow. Summary of the isolation procedure and sampling, AGEs concentration, application duration, and effect on outcome measurement proliferation and apoptosis.

Study name	Year	Isolation PC	AGEs application		Effect on outcome	
			Concentration	Duration	Proliferation	Apoptosis
Chen et al. [45]	2010	Rat–bone marrow, DGC	50, 100, 150, 200, & 400 *μ*g/ml	24 hours	↘	↗
Chen et al. [46]	2016	Rat–bone marrow, DGC	400 *μ*g/ml	24 hours	↘	↗
Jin et al. [47]	2018	Mice–bone marrow, DGC	100, 200, & 400 *μ*g/ml	24 hours	↘	↗
Kim et al. [48]	2018	Mice–bone marrow, DGC	250, 500, 600, & 750 *μ*M	24 hours	↘	
Li et al. [49]	2017	Rat–bone marrow, DGC	200 *μ*g/ml	24 hours		↗
Li et al. [50]	2012	Rat–bone marrow, DGC	50, 100, 200, & 500 *μ*g/ml	24 hours		↗
Zeng et al. [51]	2017	Rat–bone marrow	200 *μ*g/ml	48 hours	↘	↗
Wang et al. [52]	2019	Rat–bone marrow, DGC	100, 200, & 400 mg/l	12, 24, and 48 hours	Low conc. ↗, high conc. ↘	

PC: progenitor cells; ↘: decrease; ↗: increase; DGC: density-gradient centrifugation.

**Table 3:** Summary of included studies using MSCs. Summary of the isolation procedure and sampling, AGEs concentration, application duration and effect on outcome measurement proliferation and apoptosis.

Study name	Year	Isolation SC	AGEs application		Effect on outcome	
			Concentration	Duration	Proliferation	Apoptosis
Duruksu et al. [61]	2018	Rat–pancreatic islets explants	Modified collagen 10 *μ*g/cm^2^	24, 48, & 62 hours	↗	
Fang et al. [53]	2020	Human–periodontal ligament	100 *μ*g/ml	1 to 7 days	↘	↗
Kim et al. [54]	2013	Rat–bone marrow	300 *μ*g/ml	24 hours		↗
Lu et al. [55]	2012	Human–bone marrow	25, 50, 100, 200, 400, & 800 mg/l	6, 12, 24, 48, 72,& 96 hours	↘	
Sakamoto et al. [56]	2016	Rat–bone marrow	500 *μ*g/ml	7, 11, 13, 16, & 19 days	-	
Sun et al. [57]	2013	Rat–bone marrow	50, 100, 200, & 400 *μ*g/ml AOPPs	24, 48, & 72 hours	↘	
Weinberg et al. [58]	2014	Rat–bone marrow stromal cells	50, 100, 200, & 400 *μ*g/ml	16 hours		↗
Xu et al. [59]	2019	Rat–Achilles tendons	100, 200, & 400 *μ*g/ml	24 hours	↘	↗
Yang et al. [60]	2010	Rat–Bone marrow	25, 50, 100, & 200 *μ*g/ml	6, 12, & 24 hours	↘	

SC: stem cells; AOPPs: advanced oxidation protein products; ↘: decrease; ↗: increase; -: no effect.

**Table 4:** Summary of included studies using ADSCs. Summary of the isolation procedure and sampling, AGEs concentration, application duration, and effect on outcome measurement proliferation and apoptosis.

Study name	Year	Isolation SC	AGEs application		Effect on outcome	
			Concentration	Duration	Proliferation	Apoptosis
Li et al. [62]	2018	Human–adipose tissue samples enzymatically digested	100, 200, 400, 800, & 1600 *μ*g/ml	8, 12, 24, & 48 hours		↗
Li et al. [65]	2020	Mice–adipose tissue samples enzymatically digested	20, 40, 80, & 160 *μ*g/ml	1, 2, & 4 days	↘	
Wang et al. [63]	2015	Human–adipose tissue samples enzymatically digested	50, 100, 300, & 500 *μ*g/ml	24 hours		↗
Wang et al. [64]	2016	Human–adipose tissue samples enzymatically digested	300 *μ*g/ml	24 hours		↗
Zhang et al. [66]	2018	Rats–adipose tissue samples enzymatically digested	40, 80, 120, & 160 *μ*g/ml	1, 4, & 7 days	↘	

SC: stem cells; ↘: decrease; ↗: increase.

**Table 5:** Summary of included studies using NSCs. Summary of the isolation procedure and sampling, AGEs concentration, application duration, and effect on outcome measurement proliferation and apoptosis.

Study name	Year	Isolation SC	AGEs application		Effect on outcome	
			Concentration	Duration	Proliferation	Apoptosis
Fleitas et al. [67]	2018	Rat–brain tissue samples, outgrowth neurospheres	BDNF modified with 50 *μ*M GO or MGO	6 days		↗
Meneghini et al. [68]	2010	Rat–brain tissue samples, outgrowth neurospheres	25, 50, 100 *μ*g/ml	4 days	↗	
Wang et al. [69]	2009	Rat–brain tissue samples, outgrowth neurospheres	0, 50, 100, 200, & 400 mg/l	3 & 7 days	↘	
Wang et al. [70]	2011	Rat–brain tissue samples, outgrowth neurospheres	200 & 400 mg/l	3 days	↘	

SC: stem cells; BDNF: brain-derived neurotrophic factor; GO: glyoxal; MGO: methylglyoxal; ↘: decrease; ↗: increase.

## Data Availability

The data supporting this systematic review are from previously reported studies and datasets, which have been cited. The processed data are available from the corresponding author upon request.

## Abbreviations

ADSCs: Adipose tissue-derived stem cells
AGEs: Advanced glycation end-products
AOPPs: Advanced oxidation protein products
BDNF: Brain-derived neurotrophic factor
BDSCs: Blood-derived stem cells
BM: Bone marrow
CML: N(6)-carboxymethyllysine
DGC: Density-gradient centrifugation
ECM: Extracellular matrix
EPCs: Endothelial precursor cells
esRAGE: Endogenous secretory RAGE
GO: Glyoxal
HMGB1: High-mobility group box-1 protein
NAC: N-Acetylcysteine
NSCs: Neural stem cells
MAPK: MAP kinase
MGO: Methylglyoxal
MSCs: Mesenchymal stem cells
PC: Progenitor cells
PMBCs: Peripheral blood mononuclear cells
RAGE: Receptor for AGEs
ROS: Reactive oxygen species
SC: Stem cell
sRAGE: Soluble RAGE
TF: Transcription factor.
